# Applying a Pareto‐Based Efficiency Frontier to Inform Value‐Based Pricing for Gaucher Disease Therapies in Brazil

**DOI:** 10.1002/pds.70497

**Published:** 2026-10-09

**Authors:** Marcus Carvalho Borin, Ludmila Peres Gargano, Francisco de Assis Acurcio, Juliana Álvares‐Teodoro, Augusto Afonso Guerra

**Affiliations:** ^1^ Postgraduate Program in Medicines and Pharmaceutical Assistance, Department of Social Pharmacy, Faculty of Pharmacy Federal University of Minas Gerais (UFMG) Belo Horizonte Minas Gerais Brazil; ^2^ Collaborating Center for Health Technology Assessment and Excellence in Health, Faculty of Pharmacy Federal University of Minas Gerais (UFMG) Belo Horizonte Minas Gerais Brazil; ^3^ Division of Biological and Life Sciences, Visiting Associate Professor of Epidemiology Mohamed Bin Zayed University of Artificial Intelligence Abu Dhabi UAE

**Keywords:** drug pricing, drug therapy, gaucher disease, health technology assessment, pareto‐based efficiency frontier, rare diseases

## Abstract

**Purpose:**

Gaucher disease (GD) requires lifelong high‐cost treatment within the Brazilian Unified Health System (SUS). In Brazil, treatment eligibility and monitoring are defined by Clinical Protocol and Therapeutic Guidelines (PCDT), while pharmaceutical prices are regulated by the Drug Market Regulation Chamber (CMED). Exploratory price–value approaches may help make explicit the relationship between drug prices and measurable clinical benefit. We explored the applicability of a Pareto‐based Efficiency Frontier (EF) as a descriptive hematologic price–value framework for GD therapies in Brazil.

**Methods:**

An exploratory Pareto‐based EF analysis compared annual drug acquisition prices with standardized hematologic benefits, defined as hemoglobin and platelet improvements, derived from a published systematic review. Annual prices were obtained from CMED, Maximum Price of Sale to the Government (PMVG), and median public procurement prices from the Health Price Database (BPS). Prices are presented in Brazilian reais and USD adjusted by purchasing power parity. Directly dominated technologies were excluded, and all remaining directly non‐dominated therapies were connected by linear interpolation to form a Pareto‐based 12‐month reference EF. This construction retains alternatives that would be excluded by extended dominance under a conventional convex EF. The analysis was restricted to treatment‐naïve patients.

**Results:**

Annual costs ranged from approximately USD‐PPP 94900 to 763 200. Under the primary 12‐month analysis, imiglucerase was directly dominated across all three pricing scenarios, whereas taliglucerase alfa, velaglucerase alfa, and miglustat defined the Pareto‐based frontier. In the BPS hemoglobin analysis and in all three platelet analyses, taliglucerase alfa would be subject to extended dominance under a conventional convex EF but was retained as a directly non‐dominated therapy. This classification was conditional on indirect comparisons from heterogeneous evidence and the selected 12‐month hematologic point estimates. In the descriptive scenario analysis, direct dominance of imiglucerase was less stable when maximum effects across longer follow‐up durations were considered.

**Conclusion:**

This exploratory application of a Pareto‐based Efficiency Frontier describes the relationship between current price scenarios and comparative hematologic benefit for GD therapies. Findings should not be interpreted as definitive comparative value evidence, an ICER‐based optimization rule, or a stand‐alone pricing instrument. The approach may complement broader value‐based assessment alongside QALY‐based modeling, long‐term outcomes, formal uncertainty analysis, and non‐hematologic clinical dimensions.

## Plain Language Summary

Gaucher disease is a rare genetic condition that requires long‐term treatment with effective but high‐cost medicines. In this exploratory study, we compared annual treatment costs with improvements in two routinely monitored hematologic outcomes: hemoglobin and platelet counts. We used a Pareto‐based approach that retained every therapy not directly dominated by another therapy. Consequently, the resulting line was non‐convex in four analyses and retained taliglucerase alfa where a conventional convex Efficiency Frontier would exclude it by extended dominance. The analysis was based on indirect comparisons from heterogeneous studies and should not be interpreted as a complete economic evaluation, an optimization rule, or a direct pricing instrument. Rather, it provides descriptive price–benefit references that should be considered alongside quality of life, long‐term outcomes, uncertainty, and other clinical dimensions of Gaucher disease.

## Key Points

- This study applies a Pareto‐based Efficiency Frontier as an exploratory hematologic price–value approach for Gaucher disease therapies in Brazil.
- The Pareto‐based construction retains all therapies that are not directly dominated and may therefore produce a non‐convex polyline.
- In the BPS hemoglobin analysis and in all three platelet analyses, taliglucerase alfa would be subject to extended dominance under a conventional convex Efficiency Frontier but remained on the Pareto‐based frontier.
- Frontier membership and dominance status were conditional on indirect comparisons derived from heterogeneous evidence and should not be interpreted as definitive comparative value evidence.
- The approach provides descriptive price–value references and does not represent an ICER‐based optimization rule or a stand‐alone pricing instrument.

## 1 Introduction

Gaucher disease (GD) is a rare genetic disorder caused by a deficiency of the enzyme glucocerebrosidase, which leads to the accumulation of glucocerebroside in the cells of the reticuloendothelial system, particularly in the liver, spleen, and bone marrow. As a lysosomal storage disease, it presents with a variety of symptoms, including hepatosplenomegaly, anemia, thrombocytopenia, bone complications, and, in specific cases, neurological impairment [1, 2].

GD is classified into three main clinical types: Type 1 (non‐neuronopathic), Type 2 (acute neuronopathic), and Type 3 (chronic neuronopathic). Type 1 is the most prevalent form and is characterized by the absence of neurological manifestations, making it the primary focus of clinical studies and currently available therapeutic strategies. The evaluated enzyme replacement therapies (ERTs), imiglucerase, velaglucerase alfa, and taliglucerase alfa, are used primarily to treat the systemic manifestations of GD. Miglustat is indicated in Brazil for selected adults with GD Type 1 for whom ERT is not considered suitable. Its indication for progressive neurological manifestations concerns Niemann–Pick disease type C and not GD Type 3 [3].

The treatment of GD is based on two main strategies: ERT, administered intravenously, which aims to replace the deficient enzyme and primarily acts on visceral and hematological manifestations; and SRT, administered orally, which works by reducing production of the accumulated substrate by inhibiting glucosylceramide synthase. These therapeutic approaches have demonstrated clinical efficacy, especially in GD Type 1. Mistry et al. [4] specifically evaluated the SRT eliglustat rather than ERT [4, 5].

Despite their clinical efficacy, the high cost of these therapies poses challenges to healthcare systems, including the Brazilian Unified Health System (SUS), reinforcing the need for comprehensive economic evaluations to inform pricing and reimbursement decisions. Cost‐effectiveness analysis (CEA) is widely used to assess the relationship between the costs and benefits of health technologies [6]. The conventional Efficiency Frontier (EF) maps costs against specific clinical outcomes and excludes alternatives subject to direct and extended dominance. In this study, we examine a distinct Pareto‐based adaptation that retains all directly non‐dominated therapies and uses the resulting polyline as a descriptive price–benefit reference rather than as a conventional convex cost‐effectiveness frontier or optimization rule.

In Brazil, CEA is widely used to support decisions on technology incorporation in both the public (SUS) and private (Supplementary Health) sectors. However, frontier‐based price–benefit analyses are not widely used in evaluations conducted by the National Committee for Health Technology Incorporation (Conitec), the National Supplementary Health Agency (ANS), or the Drug Market Regulation Chamber (CMED). This study therefore explores the applicability of a Pareto‐based EF as a descriptive hematologic price–benefit approach for therapies currently available for Gaucher disease in Brazil.

Brazil has developed a structured regulatory and clinical framework for Gaucher disease within SUS, including official Clinical Protocol and Therapeutic Guidelines (PCDT), which define therapeutic eligibility criteria and monitoring parameters. These guidelines establish hematologic normalization, particularly hemoglobin levels and platelet counts, as core treatment goals [7]. Furthermore, Brazil has generated one of the largest longitudinal real‐world datasets on Gaucher disease treatment globally, enabling evaluation of long‐term clinical and financial impact within SUS [5]. This context provides a unique opportunity to operationalize value‐based pricing instruments such as the Efficiency Frontier within an established clinical and regulatory ecosystem.

Additionally, recent regulatory proposals have suggested incorporating Efficiency Frontier principles into Brazilian pharmaceutical pricing policy as part of a broader value‐based framework [8]. The present study is an exploratory application of a Pareto‐based adaptation in a rare‐disease setting. Any future implementation as a regulatory pricing instrument would require comparative efficacy evidence, formal uncertainty analysis, and consideration of broader clinical value dimensions beyond the hematologic outcomes examined here.

## 2 Methods

### 2.1 Study Design and Technologies Assessed

This economic evaluation used an exploratory Pareto‐based price–value analysis to compare annual drug acquisition costs with selected hematologic benefits of therapies available for Gaucher disease in Brazil. The analysis was conducted from the SUS perspective. It does not constitute a full economic evaluation incorporating utilities, quality‐adjusted life years (QALYs), long‐term survival modeling, budget impact analysis, or probabilistic uncertainty analysis.

The Efficiency Frontier was used as an exploratory Pareto‐based hematologic price–value approach within a broader value‐based pricing framework. The analysis was designed to make explicit the observed relationship between annual treatment costs and measurable hematologic benefit. It was not designed to estimate an optimal population allocation, apply an ICER‐based decision rule, or provide a stand‐alone pricing rule.

The technologies selected for this analysis were those with marketing authorization from the Brazilian Health Regulatory Agency (Anvisa) for the treatment of Gaucher disease and available for medical prescription within SUS as of January 2023. The drugs were divided into two main therapeutic groups: enzyme replacement therapy (ERT), including imiglucerase (Cerezyme), velaglucerase alfa (VPRIV), and taliglucerase alfa (Elelyso); and substrate reduction therapy (SRT), represented by miglustat (Zavesca). Eliglustat (Cerdelga), despite its regulatory approval, was not included in the analysis because it was not available through SUS during the study period. Data on the characteristics of the analyzed technologies, including mechanism of action, route of administration, recommended dosage, and approved therapeutic indications, were obtained from regulatory documents published by Anvisa.

### 2.2 Efficacy Parameters

To estimate the clinical efficacy of the selected technologies, a targeted search of the scientific literature was performed to identify updated and high‐quality systematic reviews that comparatively assessed available therapies for Gaucher disease. The systematic review conducted by Leonart et al. [9] was selected because of its comprehensiveness, methodological quality, and timeliness. It included 68 longitudinal studies, encompassing randomized clinical trials, prospective and retrospective cohort studies, and single‐arm studies, with follow‐up times ranging from 6 months to 9 years. The results were stratified between treatment‐naïve and previously treated patients to account for different therapeutic response profiles expected in each subgroup.

The methodological quality of the selected systematic review was assessed using the AMSTAR 2 tool. The review by Leonart et al. [9] met most critical domains, including prior registration in PROSPERO (CRD42021246851), dual independent assessment in study selection and data extraction, use of standardized tools for risk‐of‐bias assessment, and execution of random‐effects meta‐analyses. This supported its classification as providing moderate confidence. One limitation was the absence of a formal assessment of publication bias, although heterogeneity was explored through sensitivity analyses. The review was therefore considered sufficiently rigorous to support the exploratory clinical efficacy estimates used in this study.

Effectiveness inputs were derived from a comprehensive systematic review synthesizing clinical outcomes across enzyme replacement and substrate reduction therapies. Hemoglobin and platelet counts were selected because they were the most consistently reported and standardized outcomes across studies, and because they are guideline‐recognized surrogate hematologic outcomes routinely monitored in Gaucher disease care. Moreover, these parameters are explicitly defined as therapeutic targets in the Brazilian Clinical Protocol and Therapeutic Guidelines for Gaucher disease and represent core monitoring indicators within SUS clinical practice [7].

We acknowledge that hemoglobin and platelet counts are surrogate hematologic endpoints and do not capture all clinically meaningful dimensions of GD, such as bone involvement, hepatic and splenic volume reduction, neurological manifestations (in Type 3), or patient‐reported outcomes. We restricted our analysis to hematologic parameters because they are the only outcomes simultaneously: (i) standardized across all four therapies in the source systematic review, (ii) defined as therapeutic targets in the Brazilian PCDT for GD, and (iii) routinely monitored in real‐world SUS clinical practice [5].

The primary outcomes were defined as the mean change from baseline in hemoglobin levels (g/dL) and platelet counts (×10^9^/L) for treatment‐naïve patients. This subgroup was selected because it provides the clearest estimate of initial hematologic response after treatment initiation and reduces confounding by prior treatment exposure, stabilization, switching history, adherence patterns, and accumulated clinical trajectory. However, this restriction limits external validity because pricing and reimbursement decisions apply to the overall treated population, including prevalent patients already receiving therapy.

For each therapy, the point estimate used to position the drug on the Pareto‐based frontier (“Δ at 12 months” in Table 2) was the pooled mean difference reported by Leonart et al. [9] at the 1‐year (12‐month) follow‐up time point. The 12‐month time point was adopted because it was consistently reported across all four therapies in the source meta‐analysis and aligned with the one‐year cost denominator. The final 2025 Brazilian PCDT recommends more frequent monitoring of hemoglobin and platelet counts and was not used to define a conventional 12‐month response‐assessment horizon.

To characterize the temporal variability of the response, we additionally extracted, for each drug and outcome, the lowest and highest pooled mean differences reported in the same source meta‐analysis at any other available follow‐up time points including 6 months, 2 years, and longer durations of up to 5 to 7 years, depending on the drug. These minimum and maximum values are shown in Table 2 as the “Range across other follow‐up time points” columns and in the Figures 1, 2 and 3 as horizontal bars. They are descriptive of how the effect estimate varied across the available follow‐up time points and correspond to the lowest and highest pooled mean differences observed across the reported follow‐up time points, regardless of which specific time point produced each extreme. These ranges do not represent confidence intervals, probabilistic uncertainty, or formal assessment of the stability of frontier membership.

**FIGURE 1:**
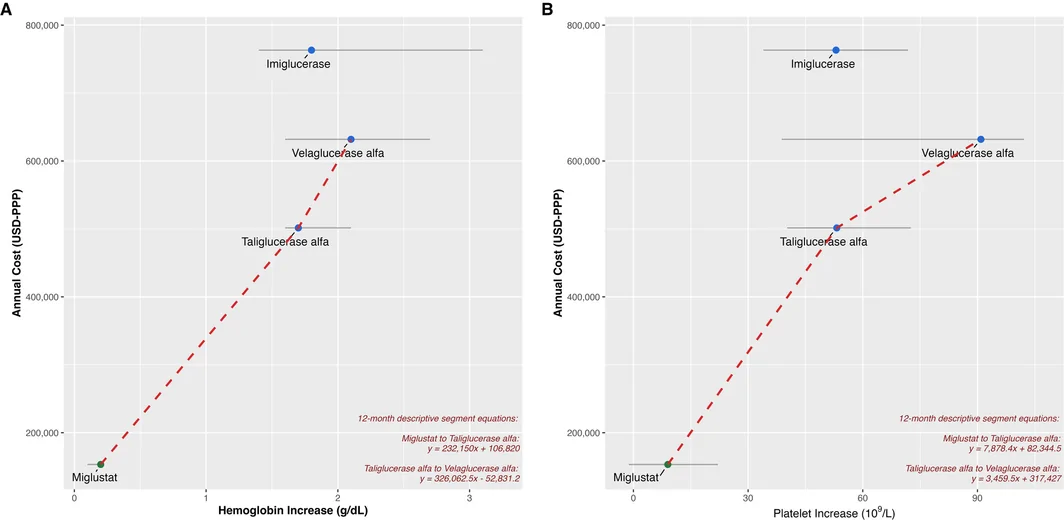
Pareto‐based Efficiency Frontier for Gaucher disease therapies using CMED prices. The graphs show the relationship between annual drug acquisition cost (USD‐PPP) and hematologic benefit for (A) increase in hemoglobin and (B) increase in platelet counts, using CMED 18% prices. Data points represent the 12‐month pooled mean effect used for frontier positioning. The dashed polyline represents the Pareto‐based 12‐month reference Efficiency Frontier connecting all directly non‐dominated therapies under the selected point estimates. This construction retains alternatives that would be excluded by extended dominance under a conventional convex Efficiency Frontier and may therefore be non‐convex. In panel B, taliglucerase alfa would be subject to extended dominance under a conventional convex construction. Horizontal bars do not represent confidence intervals or statistical uncertainty; they show the lowest and highest pooled mean differences reported across other available follow‐up time points and are included only to illustrate temporal variability. The equations are descriptive relationships between adjacent directly non‐dominated therapies; they are not ICERs, optimization rules, or stand‐alone pricing thresholds.

**FIGURE 2:**
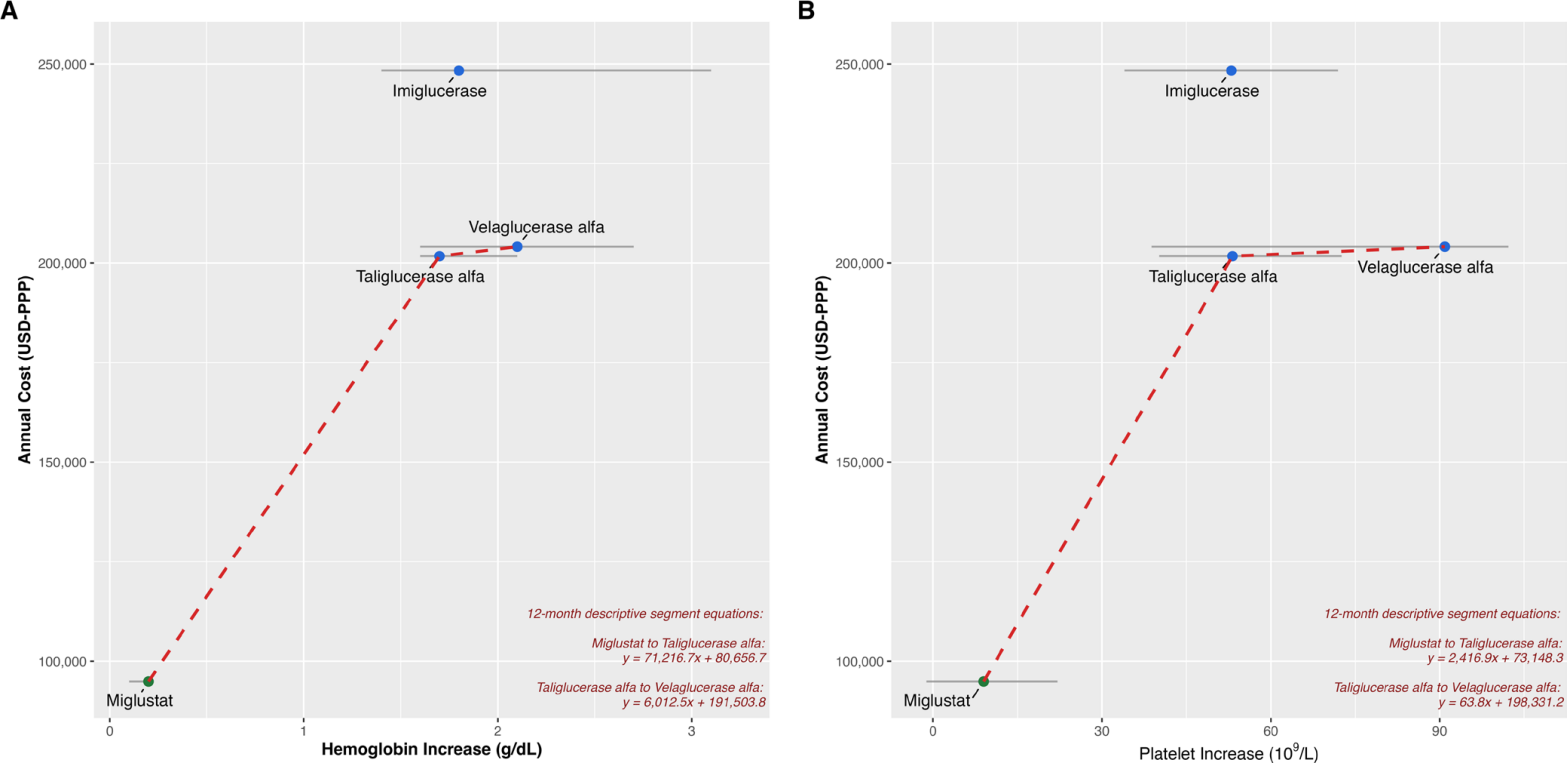
Pareto‐based Efficiency Frontier for Gaucher disease therapies using BPS prices. The graphs show the relationship between annual drug acquisition cost (USD‐PPP) and hematologic benefit for (A) increase in hemoglobin and (B) increase in platelet counts, using median BPS public procurement prices. Data points represent the 12‐month pooled mean effect used for frontier positioning. The dashed polyline represents the Pareto‐based 12‐month reference Efficiency Frontier connecting all directly non‐dominated therapies under the selected point estimates. This construction retains alternatives that would be excluded by extended dominance under a conventional convex Efficiency Frontier and may therefore be non‐convex. In panels A and B, taliglucerase alfa would be subject to extended dominance under a conventional convex construction. Horizontal bars do not represent confidence intervals or statistical uncertainty; they show the lowest and highest pooled mean differences reported across other available follow‐up time points and are included only to illustrate temporal variability. The equations are descriptive relationships between adjacent directly non‐dominated therapies; they are not ICERs, optimization rules, or stand‐alone pricing thresholds.

The use of these parameters is further supported by established scientific literature. Pastores et al. [10] highlighted the clinical relevance of normalizing hematologic parameters as a treatment goal, and the systematic review by Leonart et al. [9] synthesized changes in hemoglobin and platelet counts across the evaluated therapies. These outcomes nevertheless represent only the hematologic dimension of treatment benefit.

### 2.3 Treatment Costs

Only drug acquisition costs were considered to estimate the annual treatment cost per patient. Annual costs were estimated using price data collected on March 20, 2025, from three official Brazilian sources: the Drug Market Regulation Chamber (CMED) price, considering an 18% ICMS tax; the Maximum Price of Sale to the Government (PMVG), with 0% ICMS tax; and the median unit price reported in public procurement records between 2019 and March 20, 2025, in the Health Price Database (BPS), managed by the Ministry of Health.

We chose the median rather than the arithmetic mean because procurement transactions reported in BPS show right‐skewed distributions, with occasional extreme values (e.g., emergency or very‐low‐volume purchases) that disproportionately inflate the mean. The median therefore provides a more representative summary of the central procurement cost actually faced by SUS managers under existing legal and operational conditions. We acknowledge in the Limitations that the median does not capture the full distributional variability of national acquisition costs.

For international comparability, prices are additionally presented in USD adjusted by Purchasing Power Parity (USD‐PPP), using World Bank International Comparison Program data for the reference year. PPP‐adjusted values were selected rather than nominal exchange rate conversion to better reflect relative purchasing capacity across health systems. USD‐PPP values are provided solely for cross‐country interpretability and do not represent actual international list prices.

Unit prices were converted to estimate annual costs using the dosages recommended in the package inserts for maintenance treatment of GD. For therapies with weight‐based dosing, an average weight of 70 kg for a Brazilian adult was assumed, according to data from the Brazilian Institute of Geography and Statistics (IBGE). The following dosages were used: imiglucerase (60 U/kg every 2 weeks), velaglucerase alfa (60 U/kg every 2 weeks), taliglucerase alfa (60 U/kg every 2 weeks), and miglustat (100 mg three times daily).

### 2.4 Efficiency Frontier Calculation

The Pareto‐based Efficiency Frontier was constructed using R statistical software, version 4.2.0, with the “ggplot2” package for graphical visualization and “dplyr” for data manipulation. The analysis was conducted separately for each pricing perspective and each hematologic outcome, resulting in six exploratory analyses.

For each analysis, annual drug acquisition cost was plotted on the vertical axis and hematologic benefit on the horizontal axis. A therapy was classified as directly dominated when another available therapy had lower or equal annual cost and equal or greater hematologic benefit, with at least one strict inequality. Directly dominated therapies were excluded. All remaining directly non‐dominated therapies were ordered by increasing hematologic benefit and connected using linear interpolation to form the Pareto‐based 12‐month reference Efficiency Frontier.

This construction differs from the conventional convex Efficiency Frontier used in standard cost‐effectiveness analysis, which excludes alternatives subject to both direct and extended dominance [11, 12]. Under the convex construction, an intermediate alternative is excluded when it lies above the direct line connecting alternatives with lower and higher benefit, corresponding to a decrease in incremental cost per unit of benefit in the following segment.

The present study intentionally retained such intermediate alternatives when they were not directly dominated. Its purpose was to describe observed price–value relationships among discrete therapies rather than to identify an optimal divisible combination of treatments. One interpretation of extended dominance invokes a hypothetical population‐level allocation across strategies [13]. Pareto‐based approaches can retain directly non‐dominated strategies outside the convex hull when randomized population allocations do not correspond to the decision problem [14]. The resulting polyline may therefore be non‐convex. Its segments and equations should be interpreted as descriptive geometric relationships between adjacent directly non‐dominated therapies, not as feasible treatment mixtures, monotonically increasing ICERs, or optimization rules.

The Pareto‐based Efficiency Frontier was constructed from indirect comparisons across therapies because direct head‐to‐head comparative evidence was not available for all treatments. The underlying estimates were derived from a systematic review that included heterogeneous evidence sources, including observational and single‐arm studies, with differences in study design, baseline characteristics, patient populations, and follow‐up duration. Therefore, frontier membership and dominance status should be interpreted as conditional on the selected 12‐month hematologic point estimates and not as definitive comparative effectiveness or overall value evidence. Although a targeted search was conducted to identify the source systematic review, no independent systematic search for primary studies or update of the evidence base synthesized by Leonart et al. [9] was undertaken. Consequently, evidence published after the review may not be represented, which should be considered when interpreting the price‐oriented conclusions.

Annual drug acquisition costs correspond to 1 year of maintenance therapy. To preserve internal consistency between the cost denominator and the effect estimate, all primary Pareto‐based frontier positions were derived strictly from the pooled mean difference reported at the 12‐month follow‐up time point in the source meta‐analysis [9]. Pooled mean differences reported at any other follow‐up time point, both shorter (6 months) and longer (2 to 5–7 years, depending on the drug), were used only descriptively, to illustrate the temporal variability of response, and were not used to position therapies on the frontier.

No formal uncertainty analysis was performed around the Pareto‐based Efficiency Frontier. The ranges shown in the tables and figures describe temporal variability in pooled mean differences across other follow‐up time points reported in the source meta‐analysis. They do not represent confidence intervals, probabilistic uncertainty, or formal assessment of the stability of frontier membership. Therefore, dominance status and frontier membership should be interpreted as conditional on the selected 12‐month point estimates.

This study was based exclusively on previously published or publicly available secondary data and did not use individual patient information or identifiable personal data. This approach exempts the study from the need for approval by a research ethics committee. All sources used are properly cited, ensuring compliance with current ethical and academic standards.

## 3 Results

Among the therapies analyzed in this study, three (75%) were classified as ERT and administered intravenously: imiglucerase, velaglucerase alfa, and taliglucerase alfa. The fourth therapy (25%) was miglustat, an SRT administered orally. All these therapies are used for GD Type 1. Under the Brazilian indication, miglustat is restricted to selected adults with GD Type 1 for whom ERT is not suitable; it is not indicated for GD Type 3. Imiglucerase is the therapy with the oldest approval in Brazil (1997), followed by miglustat (2008), velaglucerase alfa (2012), and taliglucerase alfa (2013).

Price data were collected on March 20, 2025, from three official Brazilian sources: CMED 18%, PMVG, and BPS. The BPS value represents the median unit price reported in public procurement records between 2019 and the collection date. Table 1 presents the annual cost values for each therapy in the three pricing contexts.

**TABLE 1:** Annual costs of therapies in different pricing scenarios.

Drug	CMED 18%	CMED 18%	PMVG	PMVG	BPS	BPS
	(BRL)	(USD‐PPP)	(BRL)	(USD‐PPP)	(BRL)	(USD‐PPP)
Imiglucerase	1 892 758	**763 200**	1 485 248	**598 900**	615 888	**248 340**
Velaglucerase alfa	1 567 176	**631 900**	1 229 761	**495 900**	506 251	**204 130**
Taliglucerase alfa	1 243 657	**501 475**	975 899	**393 500**	500 279	**201 725**
Miglustat	380 055	**153 250**	298 229	**120 250**	235 372	**94 900**

*Note:* USD‐PPP values were calculated using the World Bank Purchasing Power Parity (PPP) conversion factor (GDP) for Brazil, reference year 2024 (1 international dollar = 2.48 BRL). PPP‐adjusted values are provided solely for cross‐country comparability and do not represent transaction prices outside Brazil. CMED and PMVG price data were collected on March 20, 2025. BPS values correspond to the median unit price identified in public procurement records between 2019 and the collection date, March 20, 2025.

Abbreviations: BPS: Health Price Database; CMED: Drug Market Regulation Chamber; PMVG: Maximum Price of Sale to the Government.

**FIGURE 3:**
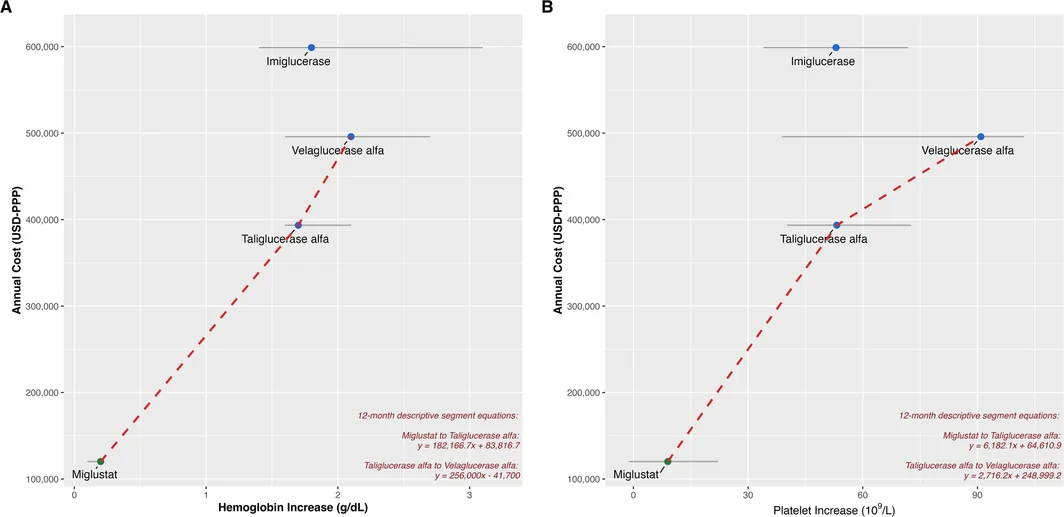
Pareto‐based Efficiency Frontier for Gaucher disease therapies using PMVG prices. The graphs show the relationship between annual drug acquisition cost (USD‐PPP) and hematologic benefit for (A) increase in hemoglobin and (B) increase in platelet counts, using PMVG prices. Data points represent the 12‐month pooled mean effect used for frontier positioning. The dashed polyline represents the Pareto‐based 12‐month reference Efficiency Frontier connecting all directly non‐dominated therapies under the selected point estimates. This construction retains alternatives that would be excluded by extended dominance under a conventional convex Efficiency Frontier and may therefore be non‐convex. In panel B, taliglucerase alfa would be subject to extended dominance under a conventional convex construction. Horizontal bars do not represent confidence intervals or statistical uncertainty; they show the lowest and highest pooled mean differences reported across other available follow‐up time points and are included only to illustrate temporal variability. The equations are descriptive relationships between adjacent directly non‐dominated therapies; they are not ICERs, optimization rules, or stand‐alone pricing thresholds.

For international comparability, costs were also presented in USD adjusted for Purchasing Power Parity (USD‐PPP), using the World Bank PPP conversion factor (GDP). For 2024, 1 international dollar ≈2.48 BRL based on available World Bank data. PPP‐adjusted values better reflect relative purchasing capacity across health systems and are more appropriate than nominal exchange rate conversions for cross‐country economic comparisons. USD‐PPP estimates are provided for interpretability and do not reflect actual transaction prices abroad.

Table 2 presents the mean efficacy values extracted from the systematic review by Leonart et al. [9], including the minimum and maximum values observed for each therapy.

**TABLE 2:** Clinical efficacy of therapies for GD.

Drug	*Δ* Hb at 12 months (g/dL)^a^	Range across follow‐up (Hb)^b^	*Δ* platelets at 12 months (×10^9^/L)^a^	Range across follow‐up (Platelets)^b^
Imiglucerase	1.8	1.4–3.1	53	34–71.9
Velaglucerase alfa	2.1	1.6–2.7	90.9	38.8–102.2
Taliglucerase alfa	1.7	1.6–2.1	53.2	40.2–72.6
Miglustat	0.2	0.1–0.2	9	(−1.2)–22.1

^a^ *Δ* at 12 months: mean change from baseline at the 1‐year (12‐month) follow‐up time point in treatment‐naïve patients, taken from the pooled mean difference reported in the source meta‐analysis [9]. This is the value used for Efficiency Frontier positioning, matching the one‐year cost denominator.

^b^ Range across other follow‐up time points: lowest and highest pooled mean differences reported in the same source meta‐analysis at any other available follow‐up time point (6 months, and longer time points up to 5–7 years, depending on the drug), shown here as a descriptive measure of the temporal variability of response. These figures are the minimum and maximum pooled mean differences (Figures 1, 2, and 3) across the follow‐up time points reported by the source review, regardless of which specific time point produced each extreme.

In treatment‐naïve patients, the mean changes from baseline used to position each therapy on the Pareto‐based 12‐month reference Efficiency Frontier corresponded to the pooled values reported by Leonart et al. [9] at the 1‐year follow‐up time point. At 12 months, mean increases in hemoglobin were 1.8 g/dL with imiglucerase, 2.1 g/dL with velaglucerase alfa, 1.7 g/dL with taliglucerase alfa, and 0.2 g/dL with miglustat. The corresponding lowest and highest pooled mean differences reported at other available follow‐up time points were 1.4–3.1, 1.6–2.7, 1.6–2.1, and 0.1–0.2 g/dL, respectively.

For platelet counts, the 12‐month mean increases were 53 × 10^9^/L for imiglucerase, 90.9 × 10^9^/L for velaglucerase alfa, 53.2 × 10^9^/L for taliglucerase alfa, and 9 × 10^9^/L for miglustat. The corresponding ranges across other follow‐up time points were 34–71.9, 38.8–102.2, 40.2–72.6, and −1.2 to 22.1 × 10^9^/L, respectively. These ranges describe temporal variability across available follow‐up time points and do not represent confidence intervals or statistical uncertainty.

Although miglustat was retained as the lowest‐cost and lowest‐benefit anchor for constructing the descriptive frontier segments, the source systematic review reported no statistically significant improvement in hematologic outcomes with miglustat among treatment‐naïve patients. Its position on the frontier therefore reflects the reported point estimates used consistently in the analysis and should not be interpreted as evidence of a statistically significant treatment effect.

Across the three pricing scenarios examined under the primary 12‐month analysis, imiglucerase was directly dominated, whereas taliglucerase alfa, velaglucerase alfa, and miglustat comprised the Pareto‐based 12‐month reference Efficiency Frontier. In the BPS hemoglobin analysis and in all three platelet analyses, taliglucerase alfa was located above the direct line connecting miglustat and velaglucerase alfa. It would therefore be subject to extended dominance under a conventional convex Efficiency Frontier. Taliglucerase alfa was retained because it was not directly dominated and because the predefined Pareto‐based construction retained all directly non‐dominated therapies. The resulting polyline was non‐convex in these four panels. These classifications were conditional on indirect comparisons derived from heterogeneous evidence and the selected 12‐month hematologic point estimates.

The set of directly non‐dominated therapies was the same across the three pricing scenarios and both hematologic outcomes. Absolute annual prices differed among CMED, PMVG, and BPS sources. However, no comparative alignment between pricing scenarios was inferred because each price source generated its own Pareto‐based reference frontier.

To illustrate the segment calculation under the PMVG scenario, a hypothetical therapy achieving a 2.0 g/dL increase in hemoglobin would correspond geometrically to an annual price of approximately USD‐PPP 470300 (BRL 1166295) on the Pareto‐based 12‐month reference polyline. This interpolation is descriptive: it does not represent a feasible treatment mixture, an ICER‐based threshold, or a regulatory pricing recommendation, and it would require reassessment if non‐hematologic outcomes, QALY‐based modeling, or formal uncertainty analysis were incorporated.

The set of directly non‐dominated therapies was the same across the three pricing scenarios and both hematologic outcomes. Absolute annual prices differed among CMED, PMVG, and BPS sources; no comparative alignment between scenarios was inferred because each source generated its own Pareto‐based reference frontier.

Linear equations were calculated for the descriptive segments of the Pareto‐based efficiency frontiers in all six analyses. The equations characterize geometric relationships between adjacent directly non‐dominated therapies under the selected 12‐month point estimates. They are not monotonically increasing ICERs, optimization rules, or stand‐alone pricing thresholds. The equations presented in the figures allow the relationships between the selected hematologic point estimates and corresponding annual prices to be reproduced.

## 4 Discussion

This study explored a Pareto‐based Efficiency Frontier as a descriptive hematologic price–value approach within a broader value‐based pricing framework for Gaucher disease therapies in Brazil. By anchoring annual drug acquisition costs to guideline‐recognized surrogate hematologic outcomes, the analysis makes explicit the relationship between price and measurable hematologic benefit using evidence available for therapies currently used in SUS.

Methodologically, the frontier used in this study should be understood as Pareto‐based rather than as the conventional convex Efficiency Frontier. It retains every therapy that was not directly dominated and may therefore be non‐convex. In the BPS hemoglobin analysis and in all three platelet analyses, taliglucerase alfa would be subject to extended dominance under the conventional convex construction. Its retention was intentional because the analysis describes observed price–value relationships among discrete therapies rather than applying an ICER‐based allocation rule.

This distinction changes the interpretation of the line segments. The equations represent descriptive geometric relationships between adjacent directly non‐dominated therapies. They do not imply that incremental cost per unit of benefit must increase monotonically and do not have the optimization interpretation associated with a conventional convex cost‐effectiveness frontier. Interpolated points on the segments should not be interpreted as feasible treatment mixtures or regulatory price thresholds.

The final 2025 Brazilian Clinical Protocol and Therapeutic Guidelines for Gaucher disease defines hematologic normalization as a treatment goal and recommends monitoring hemoglobin and platelet counts at six‐month intervals while abnormalities persist. These outcomes are routinely monitored in SUS practice and were consistently available across the therapies evaluated. In this context, the Pareto‐based frontier provides a descriptive structure for examining hematologic price–value relationships rather than a comprehensive measure of overall therapeutic value [7, 8].

The main methodological constraint of this analysis is the structural uncertainty generated by indirect comparisons across heterogeneous evidence. Although all primary Efficiency Frontier positions were anchored to 12‐month pooled mean differences, the underlying evidence was derived from studies with different designs, including observational and single‐arm studies, potentially different patient populations, and no complete set of direct head‐to‐head comparisons. As a result, the relative position of therapies on the frontier may reflect differences in study populations, baseline severity, prior clinical trajectories, and study design, rather than only true differences in comparative hematologic benefit. Frontier membership and dominance status should therefore be interpreted as exploratory and conditional on the selected inputs, not as definitive comparative evidence of superior or inferior overall value.

This limitation should also be interpreted in light of the regulatory context in which early pricing decisions are often made. For rare diseases, price‐setting and early reimbursement assessments frequently occur before mature comparative effectiveness evidence, local real‐world data, long‐term outcomes, or patient‐reported outcomes are available. In such settings, decision‐makers often need to evaluate the best available standardized evidence, including clinically meaningful surrogate outcomes and indirect comparisons, while acknowledging their limitations. The present analysis therefore illustrates how an explicit hematologic price–value anchor could be constructed using available evidence, but it should not be interpreted as replacing full health technology assessment appraisal, formal cost‐effectiveness modeling, budget impact analysis, or deliberative reassessment using local real‐world data.

Furthermore, Brazil possesses real‐world evidence infrastructure capable of monitoring long‐term Gaucher disease treatment outcomes [5]. National longitudinal data could support future recalibration of Pareto‐based price–benefit references over time within a learning health system.

Within a broader regulatory perspective, recent proposals have suggested incorporating Efficiency Frontier principles into Brazilian pharmaceutical pricing processes as part of a structured value‐based framework [15]. The present study evaluates a Pareto‐based adaptation in a rare‐disease setting and illustrates how transparent, descriptive price–benefit references may be anchored to measurable clinical outcomes [16]. These references may inform deliberation only alongside additional clinical, economic, and policy evidence; final pricing decisions remain the responsibility of the relevant decision‐makers.

Absolute annual costs were lower under the BPS procurement scenario than under the CMED and PMVG price references. BPS‐derived prices represent the median unit prices reported in Brazilian public procurement records between 2019 and the collection date, March 20, 2025, and therefore provide information about prices practiced in the public market. The median was preferred to the arithmetic mean because the procurement‐price distributions were right‐skewed and included high‐value outliers. No claim of greater alignment with a frontier‐derived threshold is made because each pricing scenario generates its own Pareto‐based reference frontier.

BPS medians provide a central summary of reported public procurement prices but do not eliminate uncertainty or represent every acquisition context. Prices may vary with time, region, procurement volume, and judicialization‐related purchases. The analysis therefore describes results at the selected median prices and does not estimate the full distribution of acquisition costs.

The results suggest that the Pareto‐based frontier can provide complementary descriptive evidence for discussions about hematologic price–value relationships, without replacing full economic evaluation or deliberative HTA. The observed relationships warrant further examination under comprehensive value frameworks incorporating non‐hematologic outcomes, long‐term survival, and probabilistic uncertainty [17].

Compared with traditional CEA, the Pareto‐based approach maps costs against a specific clinical outcome without estimating QALYs. This structural simplicity does not remove the need for valid comparative evidence and is particularly limited here because treatment effects were obtained through indirect comparisons across heterogeneous studies. The approach should therefore be considered exploratory and complementary rather than sufficient for decision‐making [18].

The exploratory application of a Pareto‐based frontier in Brazil may contribute to methodological discussions on value‐based pricing for rare diseases by making explicit the relationship between drug prices and selected measurable clinical outcomes. Its apparent simplicity should not be interpreted as reducing bias because the validity of the results remains dependent on the comparability, completeness, and uncertainty of the underlying evidence [19, 20, 21].

Although CEA is widely adopted internationally and in Brazil, its application faces several challenges, particularly regarding the establishment and application of cost‐effectiveness thresholds. The National Committee for Health Technology Incorporation (Conitec) has proposed a reference incremental cost‐effectiveness ratio threshold of approximately USD‐PPP 16129 (BRL 40000) per quality‐adjusted life year (QALY), which may reach approximately USD‐PPP 48387 (BRL 120000) per QALY under specific circumstances, including rare diseases. However, the practical application of this proposed reference remains subject to flexibility, particularly in cases of high clinical and social complexity such as Gaucher disease, creating additional challenges for the economic sustainability of SUS [22].

In this context, the SUS faces increasing difficulties due to the rising demands for high‐cost treatments, such as those for rare diseases. The budgetary impact associated with the treatment of GD highlights the need for alternative strategies for the better allocation of limited available resources. If integrated with broader HTA methods, the Pareto‐based approach could help structure transparent discussions about price–benefit relationships for high‐cost therapies [23].

In the private healthcare sector (Supplementary Health), where private operators often bear the high costs associated with treatments for rare diseases, the use of complementary approaches like the EF can also promote the financial sustainability of insurers. In this scenario, the Pareto‐based frontier may provide a structured exploratory basis for discussing hematologic price–value relationships, provided that findings are interpreted alongside broader clinical, economic, and budget‐impact evidence [24].

In Germany, EF analyses play a strategic role in specific contexts of health technology price negotiation, especially when there is an impasse between the G‐BA (Gemeinsamer Bundesausschuss) and the manufacturer after the assessment of additional benefit. Although the German system does not adopt cost‐per‐QALY as a decision criterion, these analyses are used as a preferential approach when a formal economic evaluation is necessary. They offer technical support for defining prices that are consistent with the added therapeutic value without resorting to single willingness‐to‐pay metrics, thus reinforcing the logic of efficient resource allocation in the German health system [25, 26].

Additionally, the EF approach presents significant potential to promote price competition among medicines within the same therapeutic class. This aspect can be especially relevant in contexts where biosimilar drugs or alternative therapies that are clinically equivalent but have different price structures are available. In this way, the EF can function as a practical instrument to identify concrete opportunities for cost reduction without compromising the clinical outcomes obtained by patients [27].

A challenging aspect related to the Pareto‐based EF used in this study is that it provides no explicit mechanism to limit the prices of the most effective therapies within a specific therapeutic class. As evidenced by this study, the drug velaglucerase alfa has a substantially higher cost than taliglucerase alfa, despite presenting a relatively small difference in terms of clinical efficacy. This scenario suggests that other complementary strategies, such as grouping by clinical efficacy and reference pricing, could be necessary to enhance the benefits of applying the EF in the Brazilian context [28, 29].

It is relevant to recognize that the Pareto‐based EF does not replace but may complement traditional approaches adopted by Brazilian regulatory bodies, such as CEA. The joint use of these methodologies can provide a more comprehensive and well‐founded view for decision making, especially in the face of budgetary limitations and specific clinical demands in Brazil. This methodological combination can be particularly useful in addressing rare and high‐cost diseases, thus contributing to more effective and sustainable management of health resources [30].

This study has limitations that should be considered when interpreting its results. The main limitation is the structural uncertainty generated by constructing the Pareto‐based Efficiency Frontier from indirect comparisons across heterogeneous evidence. Although the primary analysis was anchored to 12‐month pooled mean differences, the underlying evidence included studies with different designs, including observational and single‐arm studies, different patient populations, and no complete set of direct head‐to‐head comparisons. Therefore, the relative position of therapies on the frontier may partly reflect differences in study populations, baseline severity, prior clinical trajectories, and study design rather than only true differences in comparative hematologic benefit. As with populations enrolled across different randomized clinical trials used to support marketing authorization by regulatory authorities, the populations contributing to indirect cross‐study estimates cannot be assumed to be automatically comparable. Consequently, the horizontal distances used to construct the frontier should be interpreted as differences between the selected point estimates rather than as confirmed causal between‐drug effects. Frontier membership and dominance status should therefore be interpreted as exploratory and conditional on the selected 12‐month point estimates, not as definitive comparative evidence of superior or inferior overall value.

An additional methodological limitation concerns the construction of the frontier. The Pareto‐based Efficiency Frontier departs from the conventional convex construction by retaining directly non‐dominated therapies even when they are subject to extended dominance. Consequently, the resulting segment slopes are descriptive geometric relationships among the observed therapies and do not have the optimization interpretation of incremental cost‐effectiveness ratios on a conventional convex frontier. Furthermore, because the therapies are discrete clinical alternatives, points created by linear interpolation should not be interpreted as attainable treatment combinations.

Second, no formal uncertainty analysis was performed around the Pareto‐based Efficiency Frontier. The ranges displayed in the figures represent temporal variability across follow‐up periods in the source meta‐analysis and not statistical uncertainty around treatment effects or the frontier. Each pooled estimate is also subject to statistical uncertainty, as reflected by its confidence interval and between‐study heterogeneity. Neither source of uncertainty was incorporated into the frontier construction. This further compounds the instability of the resulting frontier positions. We did not perform complementary probabilistic sensitivity analysis, bootstrapping, or formal frontier‐stability analysis to test whether the relative ordering of therapies would remain stable under plausible variation in effect estimates. Consequently, the probability of frontier membership for any individual therapy cannot be estimated from the available evidence. This is particularly relevant because the central findings depend on the relative position of therapies on the frontier. Future analyses should incorporate formal uncertainty methods when individual patient data, comparative trials, or more granular evidence become available.

Third, the analysis was restricted to treatment‐naïve patients. This choice improves comparability of initial hematologic response after treatment initiation and reduces confounding by prior treatment exposure, stabilization, switching history, adherence patterns, and accumulated clinical trajectory. However, it limits external validity because pricing and reimbursement decisions apply to the overall treated population, including patients already receiving therapy. Therefore, the findings should not be extrapolated directly to all patients with Gaucher disease. Future studies should evaluate whether similar hematologic price–value relationships are observed among prevalent treated patients, switch patients, and real‐world SUS cohorts.

Fourth, the Pareto‐based Efficiency Frontier presented here reflects only hematologic value. Hemoglobin and platelet counts are clinically relevant, guideline‐recognized, and routinely monitored surrogate hematologic outcomes in Gaucher disease, but they do not capture the full clinical value of treatment. Skeletal manifestations, hepatic and splenic volume reduction, neurological manifestations, treatment burden, route of administration, quality of life, patient‐reported outcomes, and long‐term complications were not incorporated. Consequently, the price–value relationships estimated in this study should be interpreted as hematologic price–value relationships rather than as comprehensive measures of overall therapeutic value.

Fifth, cost estimates were based on publicly available procurement and list price references and included drug acquisition costs only. The BPS‐reported price used in this analysis corresponds to the median unit price in national procurement transactions between 2019 and the collection date, March 20, 2025. Although the median is less sensitive to outlier transactions, it summarizes central tendency rather than the full distributional variability of national acquisition costs, and managers in specific regions or procurement contexts may encounter higher or lower prices. CMED and PMVG references may not capture post‐incorporation discounts, confidential agreements, risk‐sharing arrangements, or budget impact constraints. Finally, PPP‐adjusted currency values were presented solely to enhance international interpretability and do not represent actual transaction prices outside Brazil.

## 5 Conclusion

This exploratory economic evaluation demonstrates how a Pareto‐based Efficiency Frontier retaining all directly non‐dominated therapies can be operationalized as a descriptive hematologic price–value approach within a broader value‐based pricing framework for high‐cost Gaucher disease therapies in Brazil. Using hematologic outcomes routinely monitored in SUS practice, the analysis characterized price–benefit relationships under the selected 12‐month point estimates.

These findings should be interpreted within the methodological scope of the study. The Pareto‐based Efficiency Frontier was constructed from indirect comparisons derived from heterogeneous evidence, did not include formal uncertainty analysis, and reflected only hematologic benefit rather than overall therapeutic value. The Pareto‐based construction was not intended to reproduce the optimization properties of a conventional convex cost‐effectiveness frontier. Therefore, frontier membership, dominance status, segment equations, and interpolated reference values should not be interpreted as definitive comparative value evidence or as stand‐alone decision rules for CMED, CONITEC, or ANS processes.

Rather, the Pareto‐based frontier may serve as one complementary descriptive input for structuring price–value discussions, alongside QALY‐based modeling, budget impact analysis, long‐term outcomes, formal uncertainty analysis, non‐hematologic clinical dimensions, and reassessment using local real‐world data. Future work should build on Brazil's longitudinal real‐world evidence infrastructure to dynamically recalibrate hematologic price–value benchmarks within a learning health system framework.

## Funding

The authors have nothing to report.

## Conflicts of Interest

The authors declare no conflicts of interest.

## Data Availability

The data that support the findings of this study are available from the corresponding author upon reasonable request.
